# Venom from Cuban Blue Scorpion has tumor activating effect in hepatocellular carcinoma

**DOI:** 10.1038/srep44685

**Published:** 2017-03-21

**Authors:** Catia Giovannini, Michele Baglioni, Marco Baron Toaldo, Matteo Cescon, Luigi Bolondi, Laura Gramantieri

**Affiliations:** 1 Center for Applied Biomedical Research (CRBA), S. Orsola-Malpighi University Hospital, Bologna, Italy; 2 Department of Medical and Surgical Sciences, University of Bologna, Bologna, Italy; 3 Department of Veterinary Medical Sciences, University of Bologna, Ozzano dell’Emilia, Bologna, Italy; 4 Department of Medical and Surgical Sciences, S. Orsola-Malpighi Hospital, University of Bologna, Bologna, Italy

## Abstract

Complementary and alternative medicine (CAM) is the term used to describe many kinds of products, practices, and systems that are not part of conventional medicine. Cancer patients usually do everything they can to combat the disease, manage its symptoms, and cope with the side effects of treatment. Unfortunately, patients who use CAM underestimate the risk of interaction with cancer therapy or worse they omit conventional therapy thus reducing the possibility of cancer remission. Herein we analyzed the effects of Vidatox 30 CH (venom extracted from the Junceus Rhopalurus scorpion) on hepatocellular carcinoma (HCC), the second leading cause of cancer-related deaths. We found out that Vidatox increases HCC proliferation and invasion whereas it does not seem to interact with sorafenib, the orally active multikinase inhibitor approved for the treatment of advanced hepatocellular carcinoma. Our results suggest that the concentration of Vidatox used in the present study has not anti-neoplastic effects and care must be taken in hiring Vidatox in patients with HCC.

Complementary and alternative medicine (CAM) is the term for diverse medical and health care systems, practices and products that are not part of conventional medical care^1^. These forms of treatment are used in addition to (complementary) or instead of (alternative) standard treatments^2^. The 2007 National Health Interview Survey reported that about 4 out of 10 adults use a CAM therapy, naming natural products and deep breathing exercises as the most commonly used treatments. Particularly CAM arouses the interest of cancer patients^3^,^4^,^5^ as supportive measures that control symptoms, enhance well-being, and contribute to their overall care. Cancer patients considering complementary and alternative therapies should discuss this decision with their oncologists because some CAM may interfere with their standard treatment or may be harmful when used with conventional therapy. Often, patients taking CAM do not inform oncologists who are not able to regard side effects of CAM substance as reason of adverse effects diagnosed in patients. CAM therapies should be evaluated with the same long and careful research process used for standard treatments. Standard cancer treatments are studied for safety and effectiveness through an intense scientific process that includes clinical trials with large numbers of patients. The National Cancer Institute (NCI) and the National Center for Complementary and Integrative Health (NCCIH) have sponsored various clinical trials that test CAM treatments and therapies in people. Some studies focused on the effects of complementary approaches used in addition to conventional treatments, and some compare alternative therapies with conventional treatments (Cancer CAM Clinical Trials-OCCAM). A small number of CAM therapies, originally considered purely alternative approaches, are finding a place in cancer treatment—not as cures, but as complementary therapies that may help patients feel better and recover faster. One example is acupuncture. According to a panel of experts at a National Institutes of Health (NIH) in November 1997, acupuncture has been found out to be effective in the management of chemotherapy-associated nausea and vomiting and in controlling pain associated with surgery^6^. However, it is not possible to evaluate all the CAM treatments due to high cost and consequently, in most cases, the side effects are unknown. Thus, CAM in oncology is still a sensitive issue due to side effects and interactions with conventional treatments and to the enormous economic impact^7^,^8^. Moreover, patients may focus on alternative treatments and omit conventional therapy thus decreasing the possibility of cancer remission^9^,^10^.

The scorpion venom is considered a natural source for cancer therapy^11^. In particular Escozul, (Labiofam) is a commercial product made from the venom of *Rhopalurus junceus*, a rare blue scorpion found only in Cuba, is considered as a potential novel cancer therapeutic^12^. In March 2011, the Cuban company Labiofam registered the product in homeopathic formula called Vidatox 30-CH that is an alcoholic solution at 33% resulting from five low molecular weight peptides extracted from the blue scorpion venom. According to Gonzalez, Vidatox was tested on more than 10,000 cancer patients with “positive results” ranging from an “improved quality of life” to a “slowing of tumor growth” (http://vidatoxromania.ro/en/what-is-vidatox/) (http://www.bt.com.bn/science-technology/2010/10/29/cuba-release-new-cancer-drug). There are no data from controlled clinical studies neither for Escozul nor for Vidatox 30-CH in refereed journals. The available information derived from interviews with patients involved or provided within the sites of alternative therapies. Essentially, scientific evidences about the biological activity of Vidatox in cancer cells are missing.

Here we evaluated the effects of Vidatox in hepatocellular carcinoma (HCC), the second leading cause of cancer mortality worldwide. We focused on HCC because no data on Vidatox efficacy are actually available in orthotopic models of HCC and because DEN-induced rat HCC recapitulates many molecular features of human HCC, as previously reported by our group. In addition, the effects of CAM could be particularly relevant in an organ involved in both drug metabolism and coagulation such as the liver.

We showed that Vidatox induces cancer cells proliferation both *in vitro* and *in vivo* whereas sorafenib induced apoptosis was unaffected by Vidatox treatment in HepG2 cells. We also found that Vidatox treated HCC cells have a high level of penetration through the matrigel-coated membrane compared with control cells.

## Results

### Vidatox induces hepatocellular carcinoma proliferation and invasion

Many cancer therapeutic agents activate the p53 pathway to induce growth arrest and apoptosis therefore, p53 status is crucial to the response of HCC to some therapies^13^. Since P53 mutations are not described in HepG2 and Snu449 cell lines, we employed them as *in vitro* models to analyze the effects of Vidatox on hepatocellular carcinoma.

**Figure 1:**
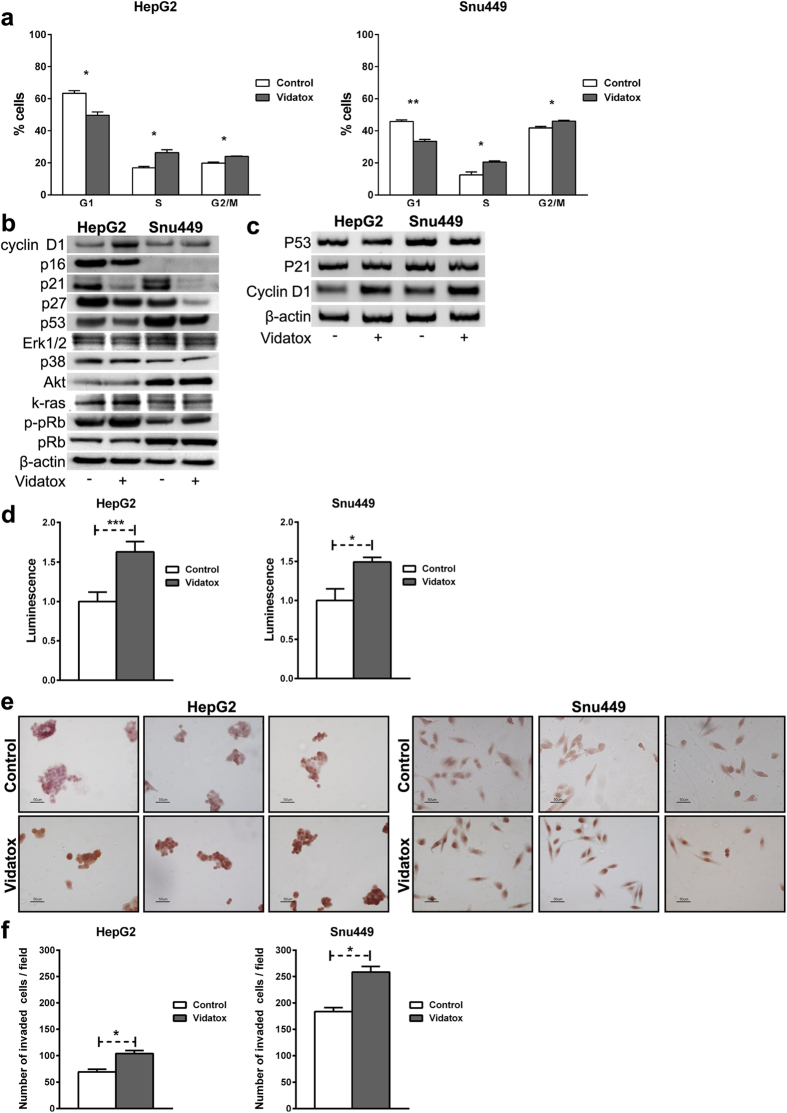
Vidatox affects the cell cycle and invasiveness ability of human HCC cells. (**a**) HepG2 and Snu449 cells were harvested 24 h after Vidatox treatment and propidium iodide staining was used to analyze cell cycle distribution. When compared to control Vidatox treated cells showed a reduction of the G1 cell population. Results are representative of three independent experiments (+/− S.E.). *P < 0.05; **P < 0.01 (by two tailed student’s t test). (**b**) Cyclin D1, p16, p21, p27, p53, Erk1/2, Akt, p38 k-Ras, phospho-pRb (p-pRb) and pRb, protein levels in control and Vidatox treated cells were analyzed by western blotting. Some cropped western blots are displayed in Supplemental Fig. 3A. Cells were exposed to Vidatox for 24 h. β Actin was used as a reference control for protein levels. (**c**) P53, P21 and Cyclin D1 gene expression were analyzed by RT-PCR in HepG2 and Snu449 cells. Cropped gels are displayed in Supplemental Figure 3B (**d**) Celltiter assay in HepG2 and Snu449 cells. ***P < 0.001 (by two tailed student’s t test). Results are expressed as the means of three independent experiments (+/− S.E.). (**e**) Ki67 expression was assessed 24 hours post Vidatox treatment by the immune-peroxidase method. Nuclei were counterstained with hematoxylin. Original magnification 20X. (**f**) Difference in invasiveness ability of HepG2 and Snu449 control and Vidatox treated cells evaluated 24 h post-Vidatox treatment. Results are the mean of three independent experiments (+/− S.E.). *P < 0.05; (by two tailed student’s t test).

To evaluate the effect of Vidatox on cell cycle regulation we firstly performed cell cycle analysis by flow cytometry. Vidatox significantly reduced cell cycle arrest at the G1/S phase transition (Fig. 1A). Then, we determined the expression levels of Cyclin D1, p53, p21, pRb, phospho-pRb, p16, p27, Akt, Erk1/2, p38 and k-Ras after Vidatox treatment for 24 h.

Vidatox treatment for 24 h triggered p53, p21, p27 and p16 down-regulation and cyclin D1 and phospho-pRb up-regulation confirming the reduced efficiency of the G1/S checkpoint in treated cells (Fig. 1B). No differences were observed in pRb, Akt, Ras and ERK1/2 protein expression upon Vidatox exposure (Fig. 1B).

P21 and p53 mRNA levels were not altered in Vidatox treated cells suggesting that Vidatox regulates their expression at post-transcriptional level. Conversely, Vidatox up-regulates Cyclin D1 mRNA expression (Fig. 1C). Both Celltiter assay and Ki-67 immuno-staining confirmed flow cytometry results (Fig. 1D,E). Moreover, Vidatox treated cells showed a higher level of penetration through the matrigel-coated membrane, compared with control cells (Fig. 1F).

**Figure 2:**
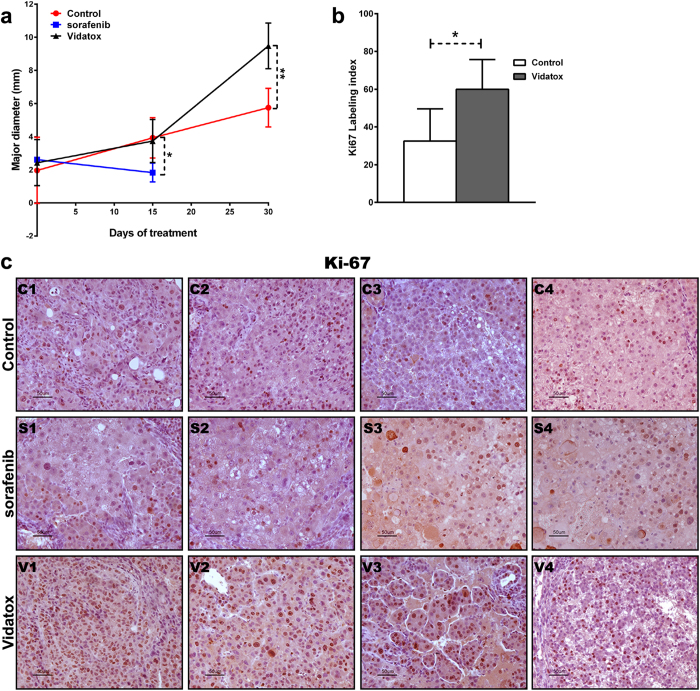
*In vivo* evidence of the role of Vidatox on tumor proliferation. (**a**) Growth curve, considering all lesions detected by imaging, showed a strong effect on nodules growth by Vidatox after 30 days of administration when compared with control. **P < 0.01 (by two tailed Student’s t test). Moreover 15 days of sorafenib treatment significantly reduced tumor proliferation p < 0.05 (by two tailed Student’s t test) compared to both control and Vidatox rats. (**b**) Quantification of growth fraction measured by Ki-67 staining in control an in Vidatox treated rat HCCs. (**c**) Immunohistochemistry analysis in four representative HCC cases of control, sorafenib and Vidatox treated rats showing Ki-67 expression. Scale bars = 50 μm. Ki-67 expression in HCCs of sorafenib treated rats was shown to appreciate the efficacy of sorafenib on cell proliferation.

**Figure 3:**
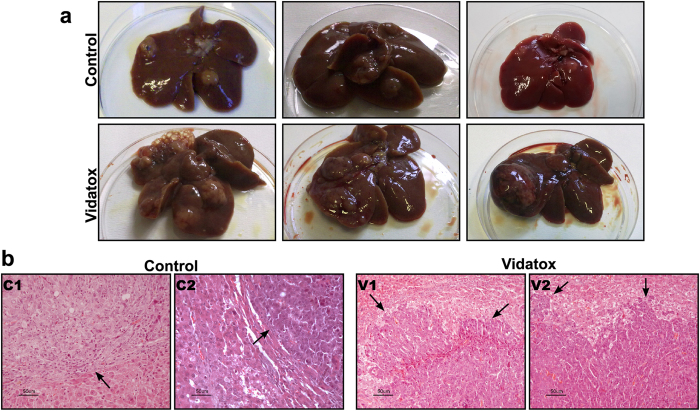
Features of the rat’s liver. (**a**) Representative photographs of liver showing a greater number of HCC nodules in Vidatox treated rats compared to controls. Moreover, a strong degeneration of the liver parenchyma is clearly visible in Vidatox group. (**b**) Hematoxylin-eosin staining of the liver of two representative control (C1-C2) and Vidatox (V1-V2) treated rats. Arrows mark the tumor fronts. In Vidatox treated rats the tumor growth fronts were invasive and irregular whereas the tumor growth fronts of control rats were found to be more regular. Original magnification 20X.

In line with the above observations in rats with HCC the treatment with Vidatox for 30 days increased tumor growth significantly more than in control rats (p = 0.01) (Fig. 2A). Indeed, HCCs proliferation was higher in rats treated with Vidatox than in control rats, as shown by immuno-staining for Ki-67 (Fig. 2B,C). Contrary, after 15 days of sorafenib exposure rat HCCs showed a much lower proliferation compared to vehicle (Fig. 2A–C). Macroscopically the rats treated with Vidatox showed a greater degeneration of the liver, a greater number of HCC nodules and larger (Fig. 3A). According to *in vitro* results in HepG2 and Snu449 cells, in the invasive edge of Vidatox treated HCCs there are more cells that penetrate the non-tumor liver tissue surrounding the HCC nodule (Fig. 3B).

Immunohistochemistry of immune cells (NK, T and macrophages) did not show significantly differences between the two groups. In detail, CD8 was highly expressed in both groups while CD56 and CD68 were only occasionally expressed in non-tumor liver both in controls and rats treated with Vidatox (Supplemental Figure 1).

### Vidatox has no pro-oncogenic effect on normal hepatic cells

**Figure 4:**
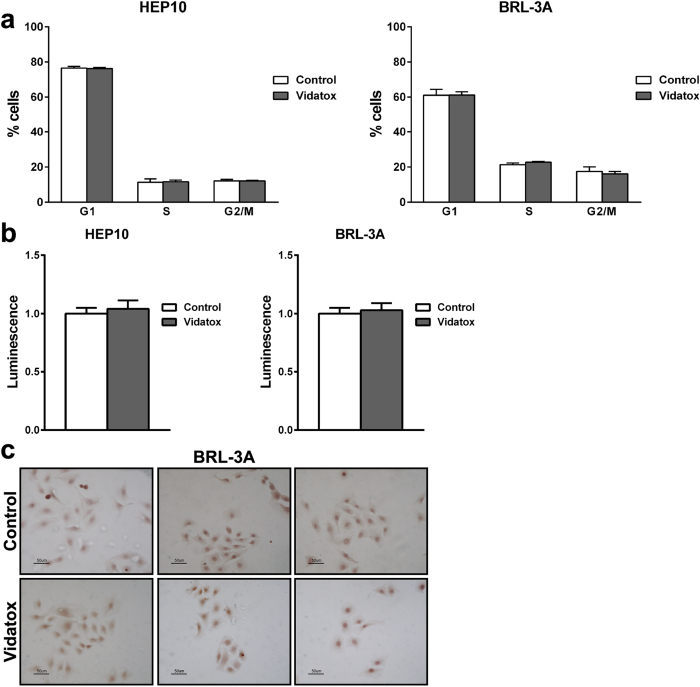
Effects of Vidatox on normal hepatic cells. (**a**) HEP10 and BRL-3A cells were harvested 24 h after Vidatox treatment and propidium iodide staining was used to analyze cell cycle distribution. Results are representative of three independent experiments (+/− S.E.). (**b**) Celltiter assay in HEP10 and BRL-3A cells. Results are expressed as the means of three independent experiments (+/− S.E.). (c) Ki67 expression was assessed 24 hours post Vidatox treatment by the immune-peroxidase method in BRL-3A. Nuclei were counterstained with hematoxylin. Original magnification 20X.

To investigate possible Vidatox pro-oncogenic effects, HEP10 suspension hepatocytes and BRL-3A fibroblasts were treated with Vidatox for 24 h. No difference was observed in cell proliferation evaluated by FACS analysis, Celltiter assay and Ki67 protein expression between control and Vidatox treated cells (Fig. 4A–C). Moreover, neither control nor fibroblasts treated with Vidatox show ability to penetrate through the matrigel-coated membrane. These results let suppose that Vidatox has no pro-oncogenic effect *in vitro* but exerts its effect only in cancer cells already affected by many cellular derangements.

### Vidatox has not synergic or enhancing effect on the anti tumoral activity of sorafenib

**Figure 5:**
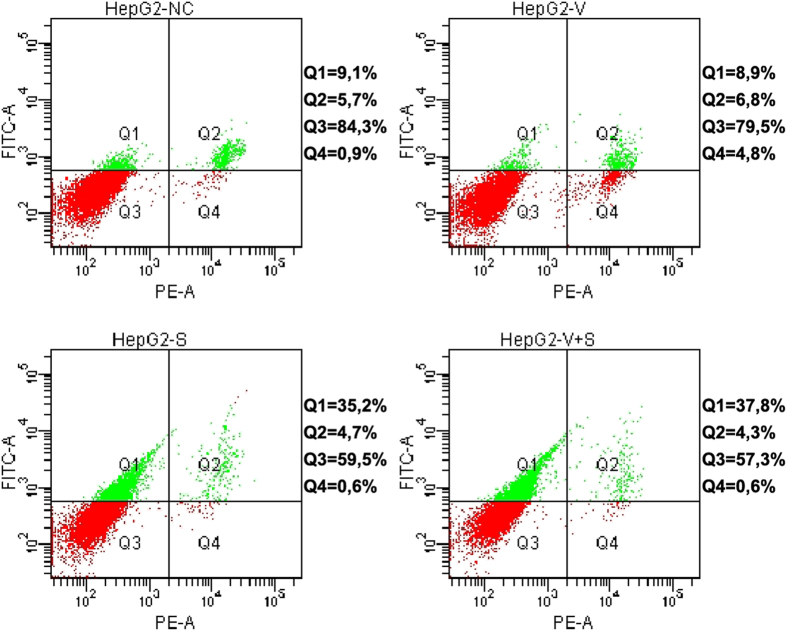
Vidatox has no effect on sorafenib induced apoptosis. After administration with Vidatox, sorafenib (4 μM) or combined treatment for 48 h HepG2 cells were labeled with Annexin V-FITC and propidium iodide. The distribution pattern of live and apoptotic cells was determined by FACS analysis. Viable cells display no Annexin and propidium iodide staining (Q3); early-stage apoptotic cells display high Annexin and low propidium iodide staining (Q1); late-stage apoptotic cells display high Annexin and high propidium iodide staining (Q2); DNA fragmentation is represented by high propidium iodide and low Annexin staining (Q4). X-axis represents propidium staining (PE) and y-axis represents FITC staining. Data are representative of at least three independent experiments. NC: negative control; V: Vidatox; S: sorafenib.

Potentially CAM products use might limit other conventional measures of the oncological treatment. The oral multikinase inhibitor sorafenib is the standard of care for advanced HCC^14^ thus, we analyzed the effect of the combined treatment sorafenib plus Vidatox on apoptosis of HepG2 cells. We found that Vidatox treatment has no effect on sorafenib induced apoptosis as evaluated by Annexin V-FITC intensity (Fig. 5).

### Effects of Vidatox on tumor angiogenesis

Targeting tumor vasculature as a cancer therapy is an established concept to benefit patients with a wide variety of tumor types because tumor angiogenesis implies a rapidly growing tumor^15^. Interestingly, VEGFR2 has been shown to transduce signals that mediate angiogenesis, vascular remodelling and cellular proliferation and its expression correlates with prognosis in HCC^16^.

**Figure 6:**
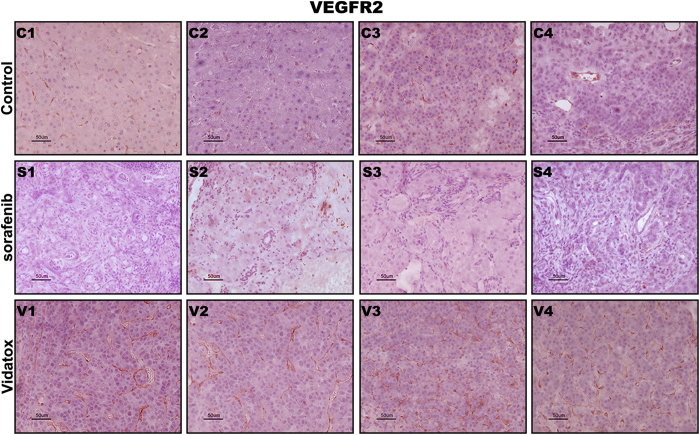
Vidatox treatment induce VEGR2 expression in rats HCC VEGFR2 expression evaluated by immunohistochemistry analysis of representative HCC cases of control (C1–C4), sorafenib (S1–S4) and Vidatox (V1–V4) treated rats. Scale bars = 50 μm. As detailed in the material and methods section VEGFR2 expression was quantified as 1 in control rats, 0 and 2 in sorafenib and Vidatox treated rats respectively.

Vidatox has been supposed to bind to tumor cells and block tumor angiogenesis (http://artemisinine.net/herbal-products/vidatox-scorpion-venom-30-ch). Thus, we analyzed VEGFR2 expression by IHC in control, sorafenib and Vidatox treated rats. In line with previous studies^17^, we found that the treatment with sorafenib (10 mg/kg/d) for 15 days significantly reduced VEGFR2 expression compared to vehicle whereas Vidatox administration increased VEGFR2 expression (Fig. 6).

### Vidatox treatment is associated with higher levels of tumor necrosis factor

**Figure 7:**
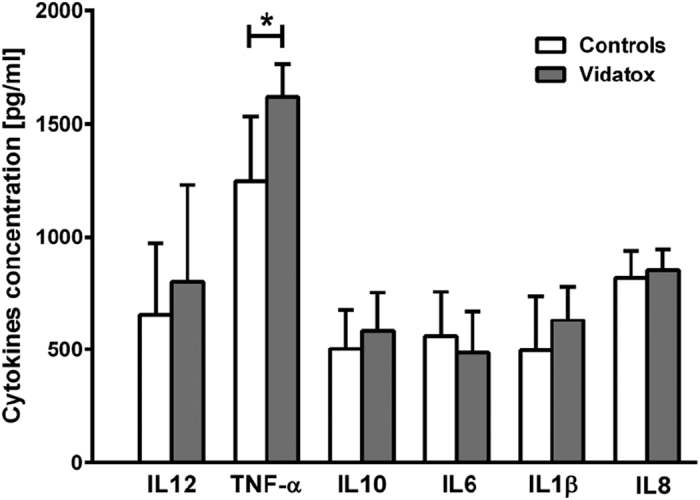
Determination of cytokines level in the sera of rat HCC. Levels of cytokines in sera were determined by a specific cytofluorimetric assay. The levels of TNFα in rats treated with Vidatox were significantly higher than in control rats (P < 0.05). Data are expressed as means (+/− S.E.). P values were derived from a two-tailed student’s t test.

Hepatocellular carcinoma arises in association with infection and chronic inflammation and exhibits extensive inflammatory infiltrates with high levels of cytokine expression in the tumor microenvironment^18^. Several such cytokines were found to serve as growth and survival factors that act on premalignant cells^19^, stimulate angiogenesis, tumor progression and metastasis, and also maintain tumor-promoting inflammation^20^. We evaluated the expression of six cytokines in serum of controls and Vidatox treated rats and found a statistical difference in the expression of tumor necrosis factor (TNF-α). Indeed TNF-α expression resulted higher in Vidatox treated rats than in control animals (Fig. 7). Since macrophage infiltrate resulted comparable in the two groups, TNF-α is likely produced by the neoplastic cell component^21^.

## Discussion

The use of complementary and alternative medicine among cancer patients is high^22^,^23^ and it appears to increase in specific populations such as breast cancer patients^24^. Usually cancer patients are aware of CAM by a family member, friends or coworkers. However, it is also important to acknowledge the influence that ethnicity may have on CAM use. For example, traditional Chinese medicine and Ayurveda are the primary source of health care in different countries. As a result, many cancer patients arrive at first consultation already using CAM therapies that are not considered “alternative” within their ethno-cultural community. The media also play a role in introducing a range of treatment options to cancer patients that may not be discussed by conventional health care providers. However, the information provided in media articles appears insufficient to assist patients with informed decision-making^25^. Unfortunately, several CAM therapies contain substance, like heavy metal, that could be harmful^26^ or may interact with conventional cancer therapies.

Among CAM the scorpion venom is considered a natural source for cancer therapy^27^. The scorpion that arouses the most interest is the Cuban Rhopalurus Junceus (Blue Scorpion), whose venom has been tested in humans suffering from cancer (http://www.escozul-cancer.com/eng/escozul-research.html). Venom is available in two different solutions: homeopathic and chilled. The homeopathic solution is called Vidatox (which is sold in Cuban pharmacies) or Trj c30 (which is the name of the product sold only to Cubans). The refrigerated (Escozul) is primarily produced in Havana from Labiofam. The main difference between the homeopathic and the refrigerated product is in the mode of application; the dosage of Vidatox is the same for all patients whereas the dosage of Escozul is personalized. Vidatox can be bought without a medical prescription and therefore its use has increased over time. There are several communications dealing with various aspects of the venom from R. Junceus, such as toxicity and pharmacology, however, the venom from this scorpion has not been studied using molecular biological approaches^28^.

Here we analyzed the effects of Vidatox in HepG2 and Snu449 HCC cell lines and we found that it induces cell proliferation. In search of a lead as to how Vidatox could enhance proliferation we analyzed the expression of well-established hallmark of cell growth, p53, p21 p16 and p27 and we found out that they were down-regulated upon Vidatox exposure^29^,^30^. Accordingly, with these results Cyclin D1 and phospho-pRb were up-regulated in Vidatox treated cells. Moreover, control cells showed a much lower proliferation than Vidatox treated ones, as shown by immuno-staining for Ki-67. In agreement with the *in vitro* data, Vidatox promotes tumor growth as demonstrated by increased tumor volume and high Ki-67 labelled growth fraction in rat HCCs without affecting innate immunity evaluated by CD8, CD56 and CD68 expression. Since these findings were rather unexpected, some rats of the same litters were treated with sorafenib, which confirmed its effectiveness in determining smaller liver nodules with haemorrhagic and necrotic aspect at sacrifice. We proved that Vidatox treatment significantly improved VEGFR2 staining *in vivo* suggesting that it plays a role in neo-angiogenesis that it’s essential for tumor growth and metastasis. It has been proven that TNF-α increases the transcription of the VEGFR2 gene (KDR) in vascular endothelial cells and the augmented expression of VEGFR2, after TNF-α stimulation, accounts for the increased cells migration^31^,^32^. Indeed Serum TNF-α levels were associated with HCC severity^33^. In line with these observations, serum TNF-α levels were higher in rats treated with Vidatox compared to controls and the invasion capabilities of HCC cells were significantly induced by Vidatox treatment. Conversely, Vidatox did not show pro-oncogenic effect neither in normal hepatocytes nor in liver fibroblasts suggesting that it exerts its effects only in tumor cells already characterized by altered pathways.

Sorafenib (Nexavar) is an orally active multikinase inhibitor approved for the treatment of hepatocellular carcinoma. Monotherapy with sorafenib prolongs overall survival and delays the time to progression in patients with advanced hepatocellular carcinoma who are not candidates for potentially curative treatment^34^. Thus, sorafenib represents an important advance in the treatment of advanced hepatocellular carcinoma and is the new standard of care for this condition^14^. Since patients with advanced cancer use CAM more often than others^35^, it is conceivable that some patients taking sorafenib may also take Vidatox thinking to improve the course of the tumor or to reduce sorafenib side effects such as hand foot syndrome. Here, we analyzed the effects of Vidatox on the sensitivity of HCC cells to sorafenib treatment *in vitro* and we found that Vidatox does not affect apoptosis induced by sorfenib as evaluated by Annexin V staining. These results together with those obtained on rats treated with Vidatox discouraged us to test the association with sorafenib *in vivo*. One major drawback of our study resides on the dosage of Vidatox used in our experiments. However, there are not previous studies that could help in dose selection. Based on our experiments here reported and in line with previous studies on the venom of scorpion Jendeki^36^, we can conclude that the concentration of Vidatox used has not anti-neoplastic effects in DENA-induced rat HCCs. Instead, in this disease as well as in HCC derived cell lines, Vidatox enhances proliferation and invasion capability. Even though these finding cannot be translated in the human setting, which is much more heterogeneous, Vidatox should be taken with caution in patients with HCC.

## Material and Methods

The methods were carried out in “accordance” with the relevant guidelines, including any relevant details.

### Culture cell lines

HepG2, Snu449 and BRL-3A cells were obtained from American Type Culture Collection (ATCC, Rockville, MD, USA) maintained in Media according to ATCC instructions. HEP10 normal hepatocytes were cultured in suspension in William’s E Medium according to ThermoFisher (Waltham, MA, USA) instructions.

### Compounds and cell death assays

Sorafenib was obtained by Bayer Healthcare (BAY 43-9006, Italy), Vidatox was purchased online. HepG2 cells were seeded into 6-well dishes and allowed to attach for 24 hours before treatments. Vidatox protein concentration, quantified by Lowry method, was 0.04 mg/ml. Two drops of Vidatox (75 μl), corresponding to 3ug of protein, were added in each well using a 100 μl pipet filter tip, whereas negative controls were obtained by adding two drops of 33% ethanol. The amount of Vidatox was established by a concentration growth inhibition study in HepG2 and Snu449 cells (Supplemental Fig. 2). Apoptosis was revealed by Annexin V-FITC (Bender Medsystems, Vienna, Austria) staining via FACS (Becton–Dickinson, Oxford, England).

### Cell proliferation analyses

HepG2, Snu449 and BRL-3A cells were synchronized, seeded in 6-well plates and then treated with Vidatox for 24 h. Both floating and adherent cells were collected and fixed with 70% ethanol. Following incubation on ice for 30 min, samples were diluted with PBS and then centrifuged at 1,200 rpm for 5 min. Pellets were re-suspended in PBS with RNase (100 μg/ml) and propidium iodide (40 μg/ml). Samples were kept at room temperature 30 min in the dark. The cell cycle distribution was analyzed using a FACScan flow cytometer. The same protocol was used for HEP10 normal hepatocytes which were not sychronizated since they are enable to proliferate.

Celltiter assay was performed according to Promega instructions (Madison, WI, USA).

### SDS-PAGE and Western blot analysis

Protein extraction and quantification were performed as previously described^37^. Primary antibodies were as follows: anti-p21 monoclonal antibody (Clone SX118, Dako, Denmark), anti-p53 monoclonal antibody (Clone DO-7, Dako), anti-cyclin D1 monoclonal antibody (clone DCS-6, Novocastra, Wetzlar, Germany), and anti-β-actin monoclonal antibody (Clone AC-40, Sigma, Saint Louis, MO, USA), anti-p27 polyclonal antibody (Becton-Dickinson), anti-p16 polyclonal antibody (Santa Cruz Biotechnology, Santa Cruz, CA, USA), anti-ERK1/2 polyclonal antibody (Cell Signaling Technology, Beverly, MA), anti-kRas polyclonal antibody (Santa Cruz Biotecnology), anti-pR polyclonal antibody (Cell Signaling Technology), anti phospo-Rb^(Ser 780)^ polyclonal antibody (Cell Signaling Technology), anti-p38 polyclonal antibody (Abcam, San Francisco, CA), anti-Akt polyclonal antibody (Cell Signaling Technology). Immunoreactivities were revealed with the EnVision dextran polymer visualization system (Dako). Membrane were washed and incubated with ECL (Amersham). Signal acquisition was done with Chemidoc scanner, (BioRad, Hercules, CA) and signals were quantified using a specific densitometric software (Image Lab, BioRad) in absorbance units after light calibration with a reference autoradiography.

### RNA analyses

Total cellular RNA was prepared using Trizol (InVitrogen, Paisley, Scotland) according to the manufacturer’s instructions. One microgram of total RNA was reverse-transcribed using Superscript II (InVitrogen). Relative gene expressions were determined by semi-quantitative end-point PCR. PCR primers were reported in Table 1.

### Cell invasion assay

Invasion assay was performed as previously described^38^.

### Animals, rats HCC induction

Fifty male Wistar rats were obtained from Harlan Italy (Udine, Italy) and were housed in an animal facility at Sant’Orsola-Malpighi Hospital (Bologna, Italy). Animals were maintained at a temperature of 20–22° and fed with a standard pellet diet ad libitum. The protocols of the experiments were approved by the Ethical Committee of the University of Bologna in accordance with European legislation. HCCs were induced in male Wistar rats (weighing 125–150 g, aged 4–5 weeks), by DENA given in their drinking water (100 mg/l weekly) for 8 weeks as previously described^39^.

### Animal’s treatment

At the end of DENA treatment animals were controlled to check HCCs development by ultrasound imaging (US) weekly. Ultrasound examinations were performed using a MyLab70 XVG (Esaote, Italy) equipped with a broadband 4–13 MHz probe. Forty-five rats developed liver nodules. As soon as HCC nodules were evident at the ultrasound imaging the experimental animals were divided into three groups each containing 15 rats: control, Vidatox and sorafenib. Sorafenib was given by oral gavages for 15 consecutive days at 10 mg/kg dose whereas 2 drops of Vidatox were given sublingual twice daily. A target lesion was identified to the first ultrasound sonography (US) and followed up by repeated US during treatment. At the end of the treatments (aged 18–20 week), animals were sacrificed under isoflurane anesthesia. The liver was removed and the upper and lower surfaces of each fixed lobe were photographed. Ultrasound findings were compared with findings at sacrifice. Again, nodules larger than 2–2,5 mm were correctly identified and nodules size was correctly estimated. Conversely, nodules less than 1,5-2 mm in size were sometimes missed. A correct estimation of liver parenchyma alteration was possible by ultrasound examination in most cases. Each tumor mass was collected and snap-frozen in liquid nitrogen or fixed in 10% formalin and paraffin embedded for histopathological analysis

### Immunohistochemistry (IHC)

The expression of VEGFR2 (Cell Signaling Technology), Ki-67 (Dako), CD8 (Biorbyt, San Francisco, CA, USA) CD56 (Abcam) and CD68 (Abcam) in rat HCCs were immunohistochemically assessed on formalin-fixed, paraffin-embedded sections. Serial 4 μm thick sections were processed for haematoxylin and eosin staining and for immunohistochemistry. Endogenous peroxidases were inhibited by incubating slides in 3% H_2_O_2_–methanol for 20 min at 4 °C. For antigen retrieval, slides were immersed in pH 6.0 citrate buffer (pH 6.0) and boiled using a microwave owen. Negative controls were obtained by omitting the primary antibody. Immunoreactivity was revealed with the EnVision system (DAKO), and diaminobenzidine (DAB) as chromogen (Sigma). Slides were counterstained in Meyer’s hematoxylin, coverslipped and examined by light microscopy. Ki-67 staining was quantified by image cytometry using Image J software (NIH, Bethesda, USA) on at least 7 randomly selected consecutive fields at 40 x and expressed as the percentage of positive nuclei over the total nuclei (Labeling index:LI). VEGFR2, CD8, CD56 and CD68 immunostaining were assessed on 15 consecutive 40X magnification fields by two independent observers (L. G., C. G.) using a validated semi-quantitative scale where 0, absence of staining; 1, staining of 5–30%; 2, staining on >30%.

### Immunocytochemistry (ICC)

HepG2, Snu449 and BRL-3A cells were seeded on sterilized coverslips and fixed in cold methanol for 10 min. Cells were then incubated with normal goat serum at room temperature for 30 min. Ki67 protein expression into the cell was assessed by using the same antibody used in IHC (Dako) followed by a HRP-rabbit EnVision system with DAB (Sigma) as chromogen. Cells were then counterstained with Mayer’s hematoxylin and mounted with DPX (BDH Chemical, Poole, UK). Negative controls were obtained by omitting the primary antibody.

### Cytokines evaluation

The standard clinical Cytometric Bead Array (CBA) (Becton–Dickinson) assessed Cytokines expression in rat serum.

### Statistical analysis

Differences between groups were analyzed using a double-sided Student t-test. P-values less than 0.05 were considered statistically significant. Statistical analyses were performed using SPSS version 19.0.

## Additional Information

**How to cite this article:** Giovannini, C. *et al*. Venom from Cuban Blue Scorpion has tumor activating effect in hepatocellular carcinoma. *Sci. Rep.* **7**, 44685; doi: 10.1038/srep44685 (2017).

**Publisher's note:** Springer Nature remains neutral with regard to jurisdictional claims in published maps and institutional affiliations.

## Supplementary Material

Supplementary Information

## Figures and Tables

**Table 1:** Primer sequences for RT-PCR.

Gene	Primers sequence (5′-3′)	Annealing T (°C)	Cycle n°	Analysis
P53 F P53 R	GGCCCACTTCACCGTACTAA GTGGTTTCAAGGCCAGATGT	57	31	RT-PCR
β ACTIN F β ACTIN R	GAGGCACTCTTCCAGCCTTC GGATGTCCACGTCACACTTC	55	26	RT-PCR
P21 F P21 R	GGAGACAGGAGACCTCTAAAGACC ACACAAGCACACATGCATCA	63	31	RT-PCR
Cyclin D1 F Cyclin D1 R	ACAAACAGATCATCCGCAAACAC TGTTGGGGCTCCTCAGGTTC	59	31	RT-PCR

† F, forward.

‡ R, reverse.
