# Corrigendum: Vidatox 30 CH has tumor activating effect in hepatocellular carcinoma

**DOI:** 10.1038/srep46920

**Published:** 2017-12-22

**Authors:** Catia Giovannini, Michele Baglioni, Marco Baron Toaldo, Matteo Cescon, Luigi Bolondi, Laura Gramantieri

Scientific Reports 7: Article number: 44685; 10.1038/srep44685 published online: 03 21 2017; updated: 12 22 2017.

The original title of our paper was inaccurate in referring to ‘Venom from Cuban Blue Scorpion’ and did not accurately reflect that we investigated the effects of the commercially-available product Vidatox 30 CH on hepatocellular carcinoma.

The title has been changed from:

“Venom from Cuban Blue Scorpion has tumor activating effect in hepatocellular carcinoma”.

to:

“Vidatox 30 CH has tumor activating effect in hepatocellular carcinoma”.

