# Supplementary material for: Venom from Cuban Blue Scorpion has tumor activating effect in hepatocellular carcinoma
Source: Sci Rep. 2017 Mar 21;7:44685. doi: 10.1038/srep44685 (PMC5359575; doi:10.1038/srep44685)
Supplement: Supplementary Information [file srep44685-s1.pdf]

Venom from Cuban Blue Scorpion has tumor activating effect in hepatocellular carcinoma

Catia Giovannini, Michele Baglioni, Marco Baron Toaldo, Matteo Cescon, Luigi Bolondi and Laura Gramantieri

SUPPLEMENTAL FIGURE LEGENDS

Supplemental Figure 1: Immunohistochemistry of CD8, CD56 and CD68 in rat livers.
(a) Immunohistochemistry analysis in two representative cases of control (C1-C2) and Vidatox (V1-V2) treated HCC showing CD8 expression. Scale bars= 50 μm. Hematoxylin-eosin staining of the same area stained for CD8 is also shown. Magnification 20X. (b) Representative pictures of non-tumor liver surrounding HCC nodules showing CD68 staining (Magnification 20X) and C56 (Magnification10X).

Supplemental Figure2: Dose-response curves.
HepG2 and Snu449 cells were treated with different volumes of Vidatox or 33% ethanol and cell proliferation was evaluated using a hemocytometer 24h post-treatment.

Supplemental Figure 3: Full length blots and gels.
(a) Some full length images of the blots shown in Figure 1b. (b) Full length images of gels shown in Figure 1c. BL; blank.

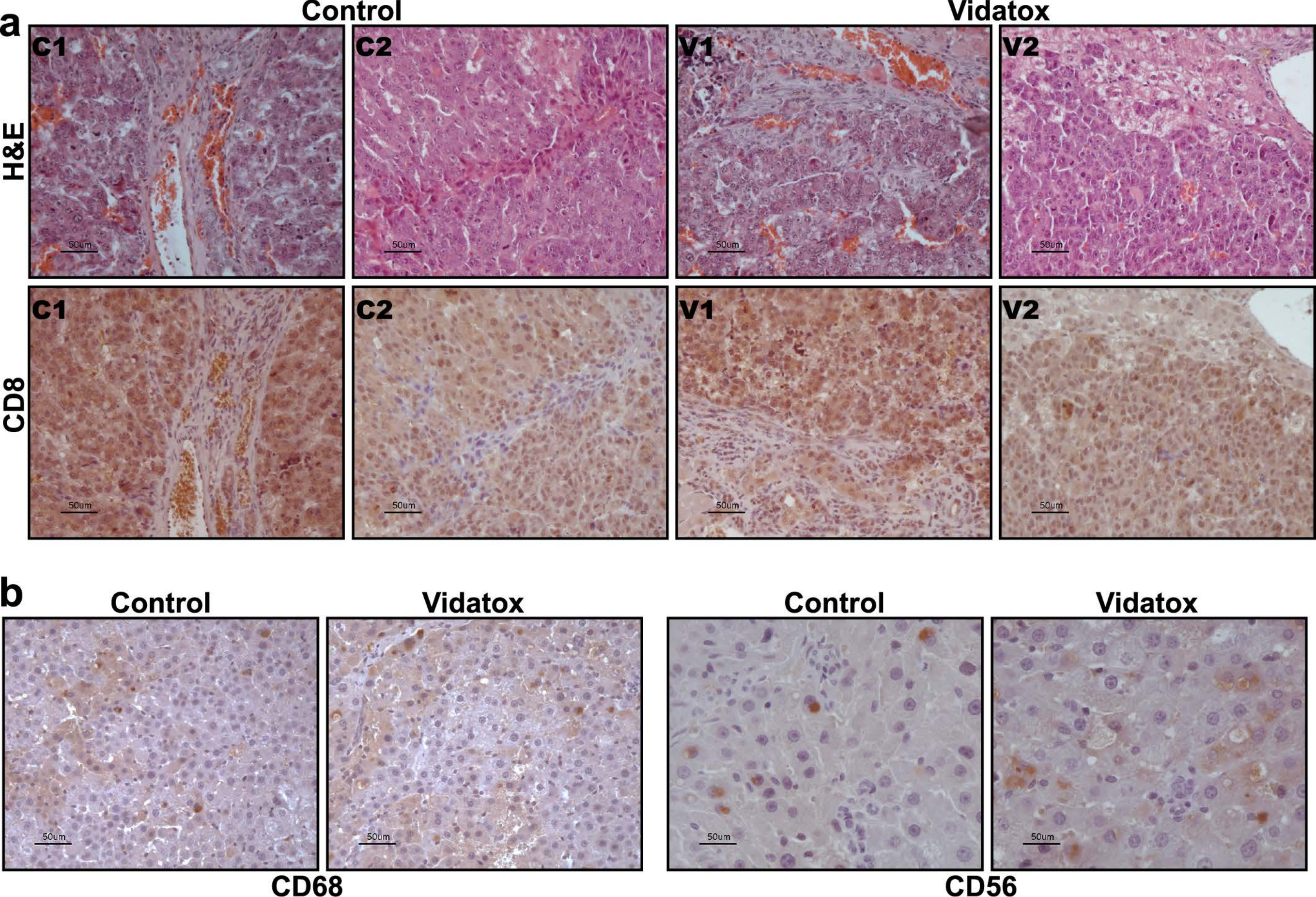
a
Control
Vidatox
H&E
CD8
C1
C2
V1
V2
50um
b
Control
Vidatox
Control
Vidatox
CD68
CD56
50um

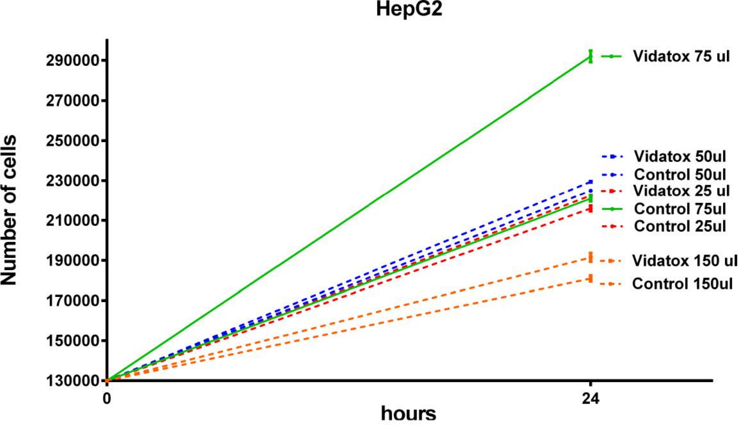
HepG2
290000
270000
250000
230000
210000
190000
170000
150000
130000
Number of cells
0
24
hours
Vidatox 75 ul
Vidatox 50ul
Control 50ul
Vidatox 25 ul
Control 75ul
Control 25ul
Vidatox 150 ul
Control 150ul

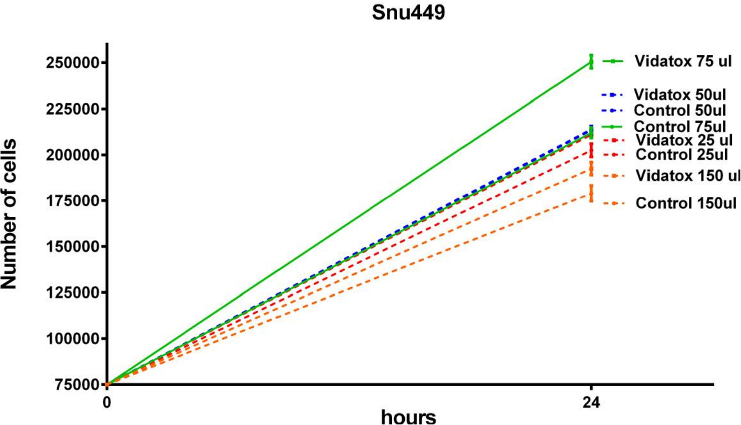
Snu449
250000
225000
200000
175000
150000
125000
100000
75000
Number of cells
0
24
hours
Vidatox 75 ul
Vidatox 50ul
Control 50ul
Control 75ul
Vidatox 25 ul
Control 25ul
Vidatox 150 ul
Control 150ul

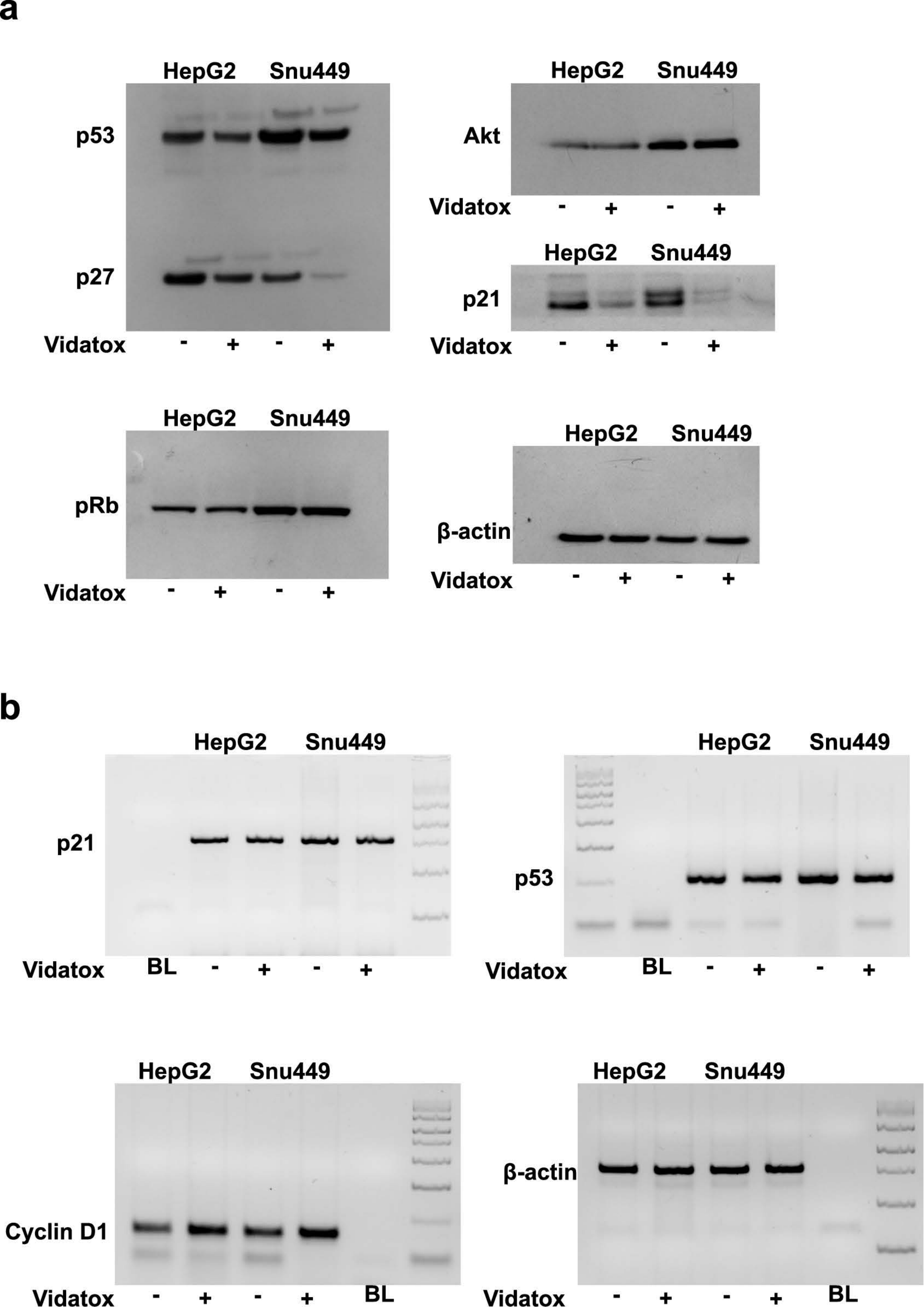
a
HepG2
Snu449
p53
p27
Vidatox
-
+
-
+
Akt
p21
pRb
β-actin
b
BL
Cyclin D1
